# Patient Health Seeking and Diagnostic Delay in Extrapulmonary Tuberculosis: A Hospital Based Study from Central India

**DOI:** 10.1155/2019/4840561

**Published:** 2019-02-03

**Authors:** Manju Raj Purohit, Rajvi Purohit, Tehmina Mustafa

**Affiliations:** ^1^Department of Pathology, R.D. Gardi Medical College, Ujjain, India; ^2^Central Clinical Laboratory, Ujjain Charitable Trust Hospital and Research Centre, Ujjain, India; ^3^Division of Global Health, Department of Public Health Sciences, Karolinska Institutet, Stockholm, Sweden; ^4^Centre for International Health, Department of Global Public Health and Primary Care, University of Bergen, Norway; ^5^Intern, R.D. Gardi Medical College, Ujjain, India; ^6^Department of Thoracic Medicine, Haukeland University Hospital, Bergen, Norway

## Abstract

**Objective:**

We aimed to investigate the awareness, health care seeking behavior, and diagnostic delay in extrapulmonary tuberculosis (EPTB) in a resource-constrained setting from Central India.

**Setting and Method:**

Questionnaire based interview of 1220 EPTB patients ≥14 years of age was conducted between July 2004 and August 2012 at Ujjain charitable Hospital, Ujjain, India.

**Results:**

Only 15% of patients had ever heard about EPTB and 2-4% knew about its prevention or treatment. Only 12% patients first sought medical advice while 49% patients practiced self-medication, 28% consulted traditional healers and 11% drug store/pharmacy. The median patient delay was 8 weeks (4.6-21.4 weeks). Majority (78%) of patients visited ≥3 health centers. Thirty-eight percent patients first visited any government health facility. Majority (97%) who first visited district and primary public health center were referred to private sector for investigations and 82% patients changed the consultation to private doctor after initial visit to public hospital. The median health system delay was 7 weeks (0.6-16.4 weeks).

**Conclusion:**

Patients had very poor awareness of EPTB. Patients were referred from public to private sector in search of diagnostic facilities. Improvement of public awareness about EPTB and better public-private partnership may contribute towards reduction in diagnostic delay.

## 1. Introduction

India accounts for 23% of the global tuberculosis burden [1]. Almost one in every six patients with pulmonary tuberculosis also exhibits extrapulmonary involvement [2, 3]. Extrapulmonary tuberculosis (EPTB) accounts for approximately 14%–40% of all tuberculosis cases [3, 4] and approximately 50% of human immunodeficiency virus- (HIV-) TB coinfected cases [1, 5, 6]. The annual incidence rate of EPTB has been shown to be increasing globally [5, 6].

Because the direct observed treatment short-term (DOTS) policy depends on the self-presentation of the patients to the health centers, proper awareness about the disease and its management is fundamental for the National Tuberculosis Control Program (NTCP). Patient awareness determines the health care-seeking behavior and practices of patients [7]. Studies on pulmonary tuberculosis have shown that delays caused by patients accounted for 77% of the total delay in treatment [8, 9]. Under the NTCP, the patient's educational programs emphasize only the cardinal symptoms of pulmonary tuberculosis; consequently, patients may be unaware of EPTB. We have previously noted that many patients with EPTB have advanced disease at clinical presentation [10, 11]. Therefore, understanding the levels of awareness of patients with EPTB and their health care-seeking behavior is necessary for developing a strategy that will improve the diagnosis and treatment of EPTB in India.

Furthermore, the diagnosis of EPTB has always been a challenge for primary health care providers. EPTB management generally requires a larger number of resource and greater clinical expertise than does that of other diseases [12, 13]. In resource-limited tuberculosis-endemic settings, the nonavailability of laboratory facilities in peripheral health centers often delays the diagnosis of EPTB [14]. The increasing incidence rates of EPTB and scant literature on the various forms of EPTB are causes of concern that should be considered while designing meaningful diagnosis and treatment guidelines under the NTCP [15].

Many studies have described the awareness levels, attitude, and health care-seeking behavior of patients with tuberculosis; however, data specifically and exclusively for patients with EPTB in Central India are currently not available. Therefore, we assessed and reported the awareness levels and health care-seeking behavior of patients with EPTB who attended a private health care facility and diagnostic delay in EPTB, in Ujjain, India.

## 2. Study Population and Methods

### 2.1. Setting

The study was conducted between July 2004 and August 2012 as a part of larger project for improving the diagnosis of EPTB at Department of Pathology, Ujjain Charitable Trust Hospital and Research Center, in Ujjain, India. The hospital serves the semiurban and rural population of Ujjain city and has a microscopy center that was established under the DOTS program of the revised NTCP (RNTCP) since 2005.

### 2.2. Patients

**Figure 1:**
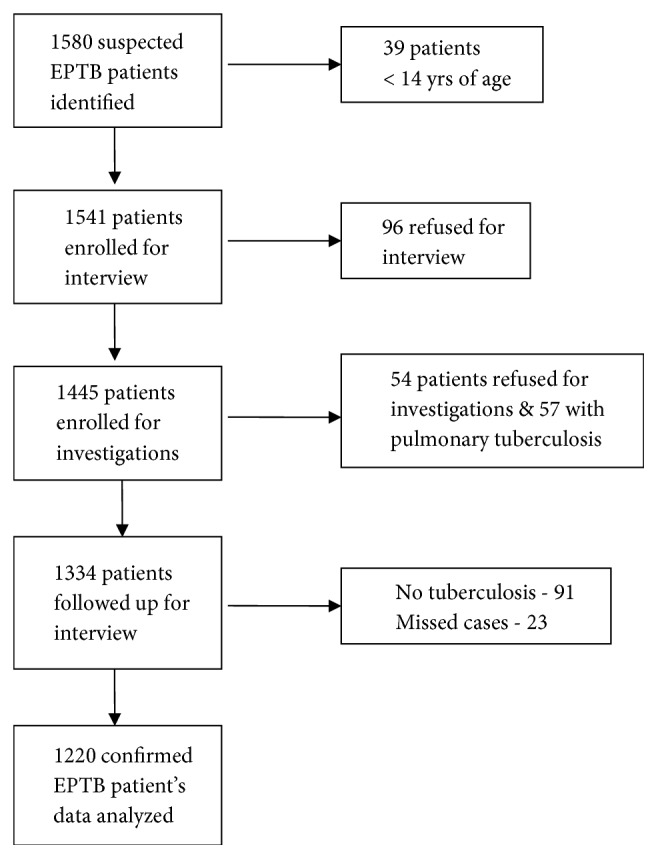
An overview of the patients enrolled in the study.

All presumptive EPTB patients referred for investigation to the Department of Pathology were prospectively enrolled. Data were collected using a predesigned questionnaire. Patients were excluded from the study if they were <14 years of age and if they were receiving treatment for tuberculosis. The diagnosis of EPTB was based on the following findings (occurring either singly or in combination) detection of acid-fast bacilli (AFB) by using the Ziehl–Neelsen (ZN) stain,* Mycobacterium tuberculosis* on culture, and nested-PCR from fluid, aspirate, or biopsy; histological or cytological features; and biochemical tests suggestive of tuberculosis with a satisfactory clinical response to treatment after an initial 8 weeks of follow-up.  Figure 1 shows the procedure for the recruitment of patients in the study. Of the 1580 patients with presumptive EPTB recruited, 1220 patients with confirmed EPTB were included in the final analysis.

### 2.3. Data Collection

A questionnaire was developed based on the literature, discussions with peers, and coauthors. The questionnaire was pretested for clarity, reliability, cultural acceptability, and ease of understanding on a few patients who were not included in the study. The questionnaire consisted of precoded closed-ended questions with a few open questions. The information on the clinical findings and results of various investigations modalities were obtained from medical records. The collected data consisted of the following: (i) sociodemographic information; (ii) clinical history (clinical form of tuberculosis, symptoms at presentation, and HIV serostatus), including personal data (smoking, alcoholism, and drug use), past history (presence of comorbidities, tuberculosis treatment, and drug regimen), family history of tuberculosis, methods of diagnosis, interval from hospital admission until diagnosis, length of hospital stay, and outcome after discharge (cured, dropout, or death as per the RNTCP guidelines); and (iii) knowledge and awareness about the cause, prevention, and treatment of EPTB that were assessed using close-ended questions. A score of 1 point was assigned for each response and total score was obtained. The median score was computed to classify patients as those with satisfactory knowledge if their total scores exceeded the median score and as those with unsatisfactory knowledge if their total scores were equal to or less than the median. The questionnaire was prepared in English, and the first author (MRP) interviewed the patients in Hindi (the local language of the study area) during the initial visit to the laboratory. Ambiguities in translations were clarified and the affected items were suitably modified by discussing with either peers or patients.

Ultrasonography, cytology, biopsy, ZN stain,* M. tuberculosis* Lowenstein Jensen culture, IS6110 based nested-PCR were performed according to the methods described previously in detail and the need of the patient work-up for diagnosis [14, 16]. HIV tests and chest X-ray were performed for all the patients included in the study. The patients and all samples were assigned a unique identification number.

*Definitions*. (1) Total delay: period from onset of the symptoms to initiation of antituberculosis treatment. (2) Patient delay: period from onset of the symptoms to first contact with any health facility. (3) Health system delay: period from first contact with health facility to initiation of antituberculosis treatment. In the present study, prolonged patient delay was defined as a patient delay of >4.2 weeks.

### 2.4. Ethical Considerations

Ethical approval was sought from the Institutional Ethics Review Committee (number 04/2004) and the regional ethical committee in Norway. Informed written consent was obtained from each patient after explaining the nature and objectives of the study. HIV testing was performed after pretest counseling and verbal consent. Patients were ensured of confidentiality, and posttest counseling was offered to patients who wished to know their results.

### 2.5. Statistical Analysis

The data were analyzed using SPSS 23 for Windows. Pearson chi-square tests were used to compare the differences in categorical variables. Multiple linear regression analyses were used to assess the association between the variables. Different patient and health system characteristics were included as dependent variables and age, sex, and site of EPTB were included as independent variables. The statistical significance was set at a* p *≤ 0.05.

## 3. Results

In the final analysis, 1220 patients who had been diagnosed with EPTB were included. The diagnoses of tuberculous lymphadenitis (n = 698), pleural tuberculosis (n = 282), abdominal tuberculosis (n = 118), tuberculous meningitis (n = 54), and other EPTB (n = 68) were confirmed according to the criterion described previously. The “other EPTB” sites included breast, genitourinary, soft-tissue, and skeletal tuberculosis. None of the patient had EPTB at multiple sites.

### 3.1. Patient Characteristics

The sociodemographic characteristics of the patients are shown in Table 1. The median age was 34 years, and 75% of the patients were between 18 and 45 years old. Most (64%) of the patients lived in rural areas, and farming or manual labor was the primary means of earning a livelihood. Tuberculous pleural effusion was more common in the male than in the female patients (male to female ratio of 1.6:1.0), while tuberculous lymphadenitis was more common in the female than in the male patients (male to female ratio 1:2.0). Diabetes was reported in only 19 patients and comorbidities were not reported in 97% patients. All the patients were HIV seronegative. Only 119 (9.7%) patients reported a history of contact with tuberculosis. Patients mainly presented with local symptoms and signs of the disease. Constitutional symptoms, either singly or in combination, were reported in 60% of patients.

### 3.2. Awareness about Tuberculosis among Patients with EPTB

Table 2(a) shows the general awareness about tuberculosis. Majority (82%–87%) of the patients were aware that the lungs are affected by tuberculosis (*p* = 0.08) with symptoms such as cough (80%–88%), fever (72%–80%), and weight loss (80%–90%) (*p* = 0.001). Most of the (67%–74%) patients responded that tuberculosis is caused by some type of “*kitandu*” (the Hindi word for germs). Nearly 50% of all the patients were not aware of the air-droplet transmission of tuberculosis or methods of tuberculosis prevention. Most of the patients (80%) knew that tuberculosis is curable (*p* = 0.08); however, only 50%–60% of the patients had heard about DOTS policy or the free test for tuberculosis in public hospitals. No significant difference was detected in the level of awareness between the male and female patients, between the rural or urban patients, or among the patients with tuberculosis at different sites.

Most (85%) of the patients were not aware about EPTB, and only 15% (194/1220) of the patients had heard about EPTB. The awareness of these 194 (15%) patients was further assessed (Table 2(b)). Very few (3%–20%) of the patients were aware of the causes, mode of transmission, symptoms and signs, prevention, treatment, and complications of EPTB.

### 3.3. Health Care-Seeking Behavior of Patients with EPTB

**Figure 2:**
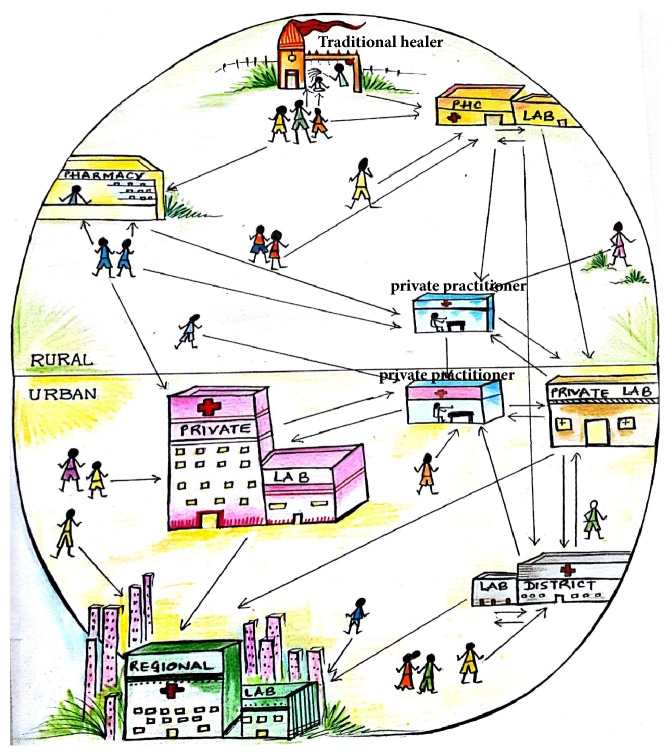
Illustration of health care practice of patients with extrapulmonary tuberculosis in rural and urban settings. The flow of patients between various care providers is unregulated, random, and haphazard. Few patients sought medical advice with the onset of disease. Many patients either practiced self-medication or first consulted any alternative care providers such as traditional healers or drug store/pharmacy for their illness. (PHC: primary health center).

Table 3 presents the health care-seeking behavior of patients with various forms of EPTB. Only 151/1220 (12%) patients first sought medical advice with the onset of disease, while many (49%) patients either practiced self-medication or first consulted alternative care providers, such as traditional healers (28%) or drug stores or pharmacists (10%), for their illness (*p* = 0.001). The patients who first sought health care from alternative care providers or self-medication and those who first sought health care from formal health care providers did not differ significantly in age, sex, residence, occupation, and education level. Majority of the patients (62%) first visited private health facility and only 38% of the patients first visited any government health facility (*p* = 0.5). Many of the patients (*p* = 0.001) visited more than three health centers. Ninety-seven percent (528/540) of the patients who first contacted any public health centers were referred to private facilities for various investigations (hematological investigation as well as cytological, histological, culture, and imaging procedures) and 82% (442/540) of patients visited private doctors after an initial visit to public hospitals (Figure 2).

### 3.4. Diagnostic Delays and Associated Factors

After presentation to the study site, 88% patients underwent three or more tuberculosis-specific investigations (chest X-ray, Mantoux test, sputum for AFB, culture) and more than two diagnostic modalities for diagnostic work-up. At least one invasive diagnostic procedure was performed in all the patients, at least two in 29% of the patients, and at least three procedures in 48/1220 (3.9%) of the patients. Diagnostic excision or incision biopsy was performed in 31% of the patients for diagnostic confirmation. Microbiological confirmation was possible in only 17% of the patients, and empirical treatment was provided to 66% of the patients.

**Figure 3:**
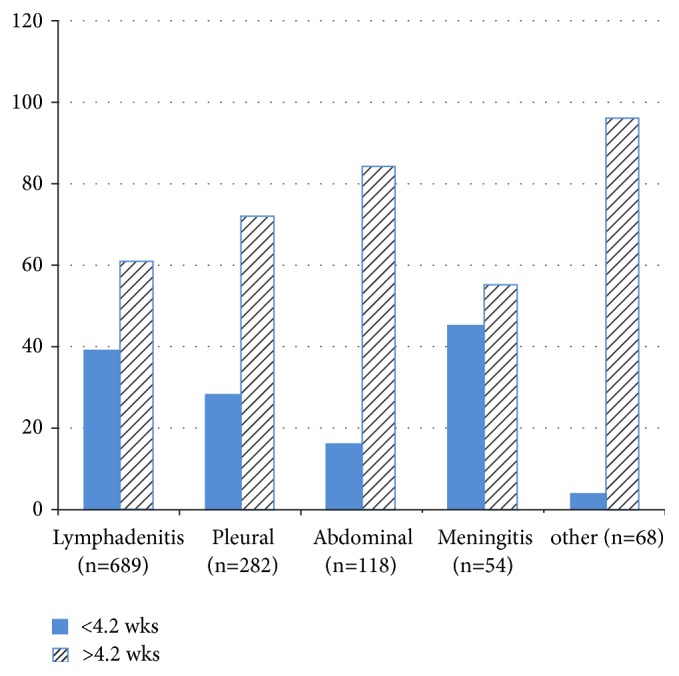
Proportion of patients with <4.2 or >4.2 weeks of total diagnostic delay in various forms of EPTB. More than 80% of patients with abdominal and other forms of EPTB had a prolonged diagnostic delay.

Table 4 shows median delay in various forms of EPTB. The median patient delay for all types of EPTB patients was 8 weeks (4.6–21.4 weeks). Patient delays of >4.2 weeks and of >8.5 weeks were observed in 72% (878/1220) and 60% of the patients, respectively. No significant differences were observed in the patients delay after adjustment for the level of awareness, age, occupation, education, and sex. The median health system delay was 7 weeks (0.6–16.4 weeks). The delay was <2 weeks in 4%, 4 weeks in 25%, 6.4 weeks in 57%, 8.5 weeks in 15%, 14 weeks in 7%, and 16.4 weeks in 2% of the patients. A longer diagnostic time (>6.4 weeks v/s <4.2 weeks) was noted in the patients who first visited public health centers after adjusting for the form of EPTB, level of awareness, age, and sex of the patients. However, the total delay differed significantly according to the site of disease. The longest delay was observed in the abdominal and other forms of EPTB while delay was the shortest in patients with meningitis (Figure 3).

## 4. Discussion

We recorded the awareness for EPTB and health care-seeking behavior across the spectrum of EPTB patients. Our results revealed very scarce awareness regarding EPTB among the patients from Central India. Patients were referred to multiple health facilities and from public to private sector for diagnosis, which resulted in diagnostic delays and increased morbidity.

The findings revealed an extensive awareness gap regarding EPTB (only 15% of the patients were aware) compared with pulmonary tuberculosis (86% of the patients were aware); this finding represents a major cause of concern for the NTCP. The lack of awareness may contribute to patient delay and initiation of treatment [17]. Awareness regarding EPTB was unsatisfactory even among urban and educated patients, which is contrary to the findings for pulmonary tuberculosis [18–20]. The levels of awareness of patients regarding tuberculosis in general and pulmonary tuberculosis were similar to those reported in other studies [20, 21]. Majority of the patients first either practiced self-medication or visited traditional healers; these findings were consistent with those of other studies from rural areas [22, 23]. Such health care-seeking behavior of patients causes complications [10, 20]; this finding emphasizes the need for improved public awareness about EPTB [3, 24].

Apart from the patient delay, long health system delays (0.6–16.4 weeks) in EPTB were observed. Most patients visited at least three medical facilities before receiving diagnosis and treatment. Various contributing factors such as atypical presentation, site of disease, lack of laboratory facilities, high cost of reagents and equipment, lack of faith or access to the public laboratories, stage of disease, complications at the time of presentation, and the need for surgical interventions can cause health system delay [17, 25–27]. These factors reaffirm the inherent complexity of the EPTB diagnosis; EPTB is further complicated by health care providers who offer empirical treatment [10]. In our study, 83% did not receive microbiological confirmation and 66% of these patients received empirical treatment, which could result in excess treatment, resulting in antibiotic resistance and increased morbidity and mortality.

A crucial finding of our study is that patients with EPTB were mainly referred to private health care centers for diagnoses. This is contrary to the findings for pulmonary tuberculosis where private patients are referred to the public health care centers for diagnosis and treatment [26, 28]. This could be due to the lack of diagnostic facilities for EPTB at the peripheral and district-level public health centers [14]. The referral practice for diagnosis contributes to treatment delays in EPTB [29]. Furthermore, patients often cannot afford to pay for diagnostic tests in private laboratories and many are lost to follow-up [30]. Our results emphasize the need to understand the diagnostic processes and providers practices as well as determine methods to overcome diagnostic delays in centralized laboratories with infrastructure for EPTB diagnosis in India.

The results highlight the need to incorporate suitable interventions in the NTCP for minimizing diagnostic delays in EPTB. Education and motivation of patients, regular training programs for primary health care providers for early recognition of various EPTB symptoms and early referral, and involvement of private sector in tuberculosis control are essential.

Considering the study design, the study has some limitations. The patients' inability to recall the dates and entire pathway of onset of symptoms and disease could lead to the collection of suboptimal information. However, experienced doctor (MRP) interviewed patients and local calendar, major national days, and festivals were used to determine patients' perceived date of onset of symptoms. Focus group discussion would have been a more suitable method than interviews to identify beliefs and attitudes towards EPTB. Only 17% of patients had microbiologically confirmed diagnosis of EPTB, and the rest of patients were categorized as EPTB based on the response to treatment at 8 weeks. This could have led to the inclusion of some non-EPTB cases. However, this bias was minimized by meticulous follow-up of patients. Because this study was based on a single private hospital, the findings cannot be generalized to the community. The effects of social, ethnic, and economic factors must be considered before extrapolating our results to all patients with EPTB in our region. Despite these concerns, the study provides baseline information regarding the awareness and health care-seeking behavior of patients with EPTB in private referral hospital settings.

## 5. Conclusion

This study highlights the inadequate awareness of EPTB in patients with unsatisfactory health care-seeking behavior and care-provision. The patients with EPTB were referred from public to private health care centers owing to a lack of diagnostic facilities in the public sector. The NTCP in India should have plans for increasing the awareness regarding EPTB, and the NTCP should collaborate with the private sector for the early diagnosis and better control of EPTB.

## Figures and Tables

**Table 1:** Sociodemographic characteristics of extrapulmonary tuberculosis patients (N=1220).

Sociodemographic characteristics	n (%)
Age (years)	
14-20	505 (41.3)
21- 40	513 (42)
>40	202 (16.5)

Gender	
Male	517 (42.3)
Female	703 (57.6)

Origin	
Rural	777 (63.6)
Urban	443 (36.3)

Occupation	
Farmer/laborer	559 (45.8)
Student	205 (16.8)
Unemployed	186 (15.2)
Employed	270 (22.1)

Education	
Illiterate	168 (13.7)
middle/secondary	724 (59.3)
Degree	328 (26.8)

Co-morbidities	
Yes	37 (3)
No	1183 (96.9)

**Table:** (a) Awareness about general aspects of tuberculosis as perceived by extrapulmonary tuberculosis patients (N =1220)

Variable n (%)^*∗*^	Tuberculous lymphadenitis (n=698)	Pleural tuberculosis (n=282)	Abdominal tuberculosis (n=118)	Tuberculous meningitis (n=54)	Other sites (n=68)	P value
*Organ affected*						
Lung	454 (65)	161 (57)	58 (49)	31 (57)	40 (59)	0.08
Lung & other organs	146 (21)	74 (26)	39 (33)	14 (26)	16 (23)	
Only other organ	1	-	1	-	2	
Do not know	98(14)	47(17)	20 (17)	09 (17)	10 (15)	

*Symptoms *						
Cough of >2weeks	610 (87)	242 (86)	98 (83)	48 (89)	55 (81)	0.001
Fever of >2 weeks	560 (80)	224 (79)	88 (74)	39 (72)	51 (75)	
Weight loss	629 (90)	230 (81)	95 (80)	45 (83)	55 (81)	
Haemoptysis& others	45 (6)	29 (10)	23 (19)	12 (22)	34 (50)	
Do not know	76 (11)	28 (10)	14 (12)	07 (13)	13(19)	

*Causes*						
Evil eye & curse	67 (9)	24 (8)	10 (8)	05 (9)	06 (9)	0.99
Germs	498 (71)	189 (67)	84 (71)	40 (74)	49 (72)	
Do not know	148 (21)	56 (20)	19 (16)	10 (18)	12 (18)	

*Mode of transmission *						
Air droplet	479 (69)	175 (62)	82 (69)	32 (59)	47 (69)	0.69
Other	85 (12)	42 (15)	16 (13)	07 (13)	10 (15)	
Do not know	158 (23)	60 (21)	29 (24)	17 (31)	14 (20)	

*Prevention*						
Yes	330 (47)	122 (43)	54 (46)	25 (46)	32 (47)	0.863
Do not know	187 (27)	67 (24)	36 (30)	14 (26)	19 (28)	

*Treatable *						
Yes	570 (82)	228 (81)	94 (80)	44 (81)	54 (79)	0.08
Do not know	48 (7)	17 (6)	10 (12)	02 (4)	04 (6)	

Know about DOTS policy	411 (59)	146 (52)	74 (62)	28 (52)	32 (47)	0.48

Heard of EPTB	94 (13)	46 (16)	28 (24)	12 (22)	14 (20)	0.91

**Table:** (b) Awareness about extrapulmonary tuberculosis among patients who had ever heard of EPTB (N=194)

Variable	number	percentage
Cause of EPTB	14	7.2
Symptoms/signs	19	9.7
Mode of transmission	09	4.6
Prevention		
Yes	06	3.7
Do not know	05	3.1
Treatment		
Yes	03	1.8
No	03	1.8
Duration of Treatment	19	12
Complication/seriousness of disease	32	20

**Table 3:** Health seeking practices of patients with various forms of extrapulmonary TB (N=1220).

Variable n (%)	Tuberculous lymphadenitis (n=698)	Pleural tuberculosis (n=282)	Abdominal tuberculosis (n=118)	Tuberculous meningitis (n=54)	Other sites (n=68)	*P* value
Perceived seriousness of illness						
Yes	322 (46)	102 (36)	65 (55)	32 (59)	23 (33)	0.001

*First treatment*						
Self-medication	334 (48)	138 (49)	63 (53)	29 (54)	39 (57)	0.001
Non-allopathic/						
traditional healer	225 (32)	67 (24)	32 (27)	04 (07)	10 (15)	
Pharmacy	76 (11)	28 (10)	09 (07)	05 (09)	10 (15)	
Allopathic	63 (13)	49 (17)	14 (12)	16 (27)	09 (13)	

*First medical facility visited*						
Regional hospital	08 (01)	02 (0.7)	00	00	02 (03)	0.579
District hospital	110 (16)	40 (14)	16 (14)	07 (13)	10 (15)	
Primary health care	135 (19)	70 (25)	31 (26)	14 (26)	15 (22)	
Private Hospital	445 (64)	170 (60)	70 (60)	33 (61)	41 (60)	

No. of facilities visited						
1	17 (2)	08 (2)	02 (2)	02 (4)	00	0.001
2	198 (28)	23 (8)	12 (11)	04 (8)	04 (6)	
3-4	429 (61)	173 (61)	84 (71)	45 (83)	34 (50)	
>4	54 (7)	78 (28)	20 (17)	03 (5)	30 (44)	

**Table 4:** Patient and health system delays in various forms of extrapulmonary tuberculosis (N=1220).

Median time *(weeks)* for Delay#	Lymphadenitis (n=698)	Pleural (n=282)	Abdominal (n=118)	Meningitis (n=54)	Other (n=68)
Patient	9.4	6.1	14.2	5.1	18

Health system	6.2	7.1	13	1.2	16.4

#p-value is significant (0.04) for the health system delay after controlling for the site of the EPTB.

## Data Availability

Detailed data supporting the results can be available from the authors on request.
